# Regulatory Factors for tRNA Modifications in Extreme- Thermophilic Bacterium *Thermus thermophilus*

**DOI:** 10.3389/fgene.2019.00204

**Published:** 2019-03-08

**Authors:** Hiroyuki Hori

**Affiliations:** Department of Materials Sciences and Biotechnology, Graduate School of Science and Engineering, Ehime University, Matsuyama, Japan

**Keywords:** methylation, RNA modification, thermophile, *Thermus thermophilus*, tRNA

## Abstract

*Thermus thermophilus* is an extreme-thermophilic bacterium that can grow at a wide range of temperatures (50–83°C). To enable *T. thermophilus* to grow at high temperatures, several biomolecules including tRNA and tRNA modification enzymes show extreme heat-resistance. Therefore, the modified nucleosides in tRNA from *T. thermophilus* have been studied mainly from the view point of tRNA stabilization at high temperatures. Such studies have shown that several modifications stabilize the structure of tRNA and are essential for survival of the organism at high temperatures. Together with tRNA modification enzymes, the modified nucleosides form a network that regulates the extent of different tRNA modifications at various temperatures. In this review, I describe this network, as well as the tRNA recognition mechanism of individual tRNA modification enzymes. Furthermore, I summarize the roles of other tRNA stabilization factors such as polyamines and metal ions.

## Introduction

*Thermus thermophilus* is an extreme-thermophilic bacterium isolated from Mine Hot Spring in Japan that can grow at a wide range of temperatures (50–83°C) (Oshima and Imahori, 1974). This bacterium can grow under aerobic conditions and possesses only about 2200 genes. Therefore, *T. thermophilus* strain HB8 was selected as a model organism in the Structural-Biological Whole Cell Project in Japan (Yokoyama et al., 2000). A method for preparing gene disruptant strains of *T. thermophilus* has been established (Hoseki et al., 1999; Hashimoto et al., 2001). Furthermore, both expression vectors for *T. thermophilus* proteins in *Escherichia coli* cells and gene disruption vectors are available from RIKEN Bio Resource Center^1^. Today, *T. thermophilus* is one of the most studied thermophiles.

Transfer RNA is an adaptor molecule required for the conversion of genetic information encoded by nucleic acids into amino acid sequences in proteins. To date, more than 100 modified nucleosides have been found in tRNA from the three domains of life bacteria, archaea, and eukaryotes (Boccaletto et al., 2018). Modified nucleosides in tRNA primarily function in various steps of protein synthesis such as stabilization of tRNA structure (Motorin and Helm, 2010; Lorenz et al., 2017), codon-anticodon interaction (Takai and Yokoyama, 2003; Suzuki and Numata, 2014; Agris et al., 2018), prevention of frame-shift errors (Björk et al., 1989; Farabaugh and Björk, 1999; Urbonavicius et al., 2001), recognition by aminoacyl-tRNA synthetases (Muramatsu et al., 1988; Perret et al., 1990; Ikeuchi et al., 2010; Mandal et al., 2010), and recognition by translation factors (Aström and Byström, 1994). In other words, living organisms cannot synthesize proteins efficiently or correctly without tRNA modifications.

**FIGURE 1:**
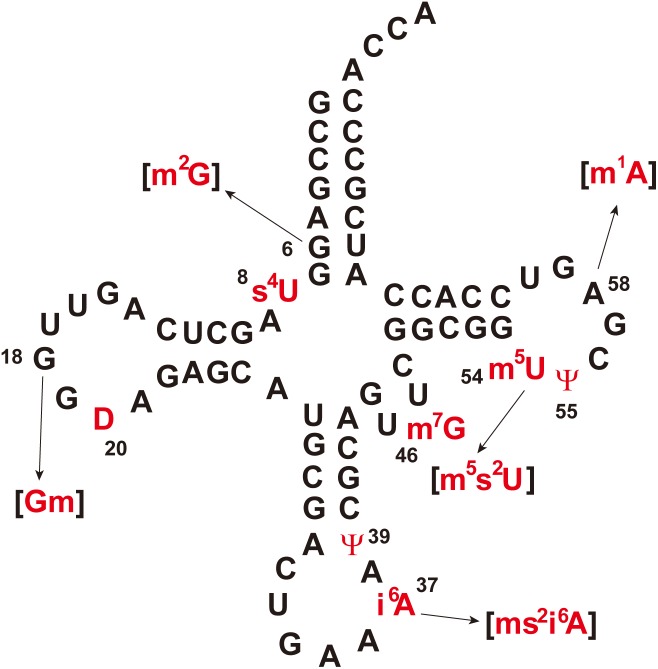
Cloverleaf representation of *Thermus thermophilus* tRNA^Phe^. Modified nucleosides are colored in red and numbers indicate the modification positions. Parentheses indicate that the modification is partial. The abbreviations of modified nucleosides are given in Table 1.

**FIGURE 2:**
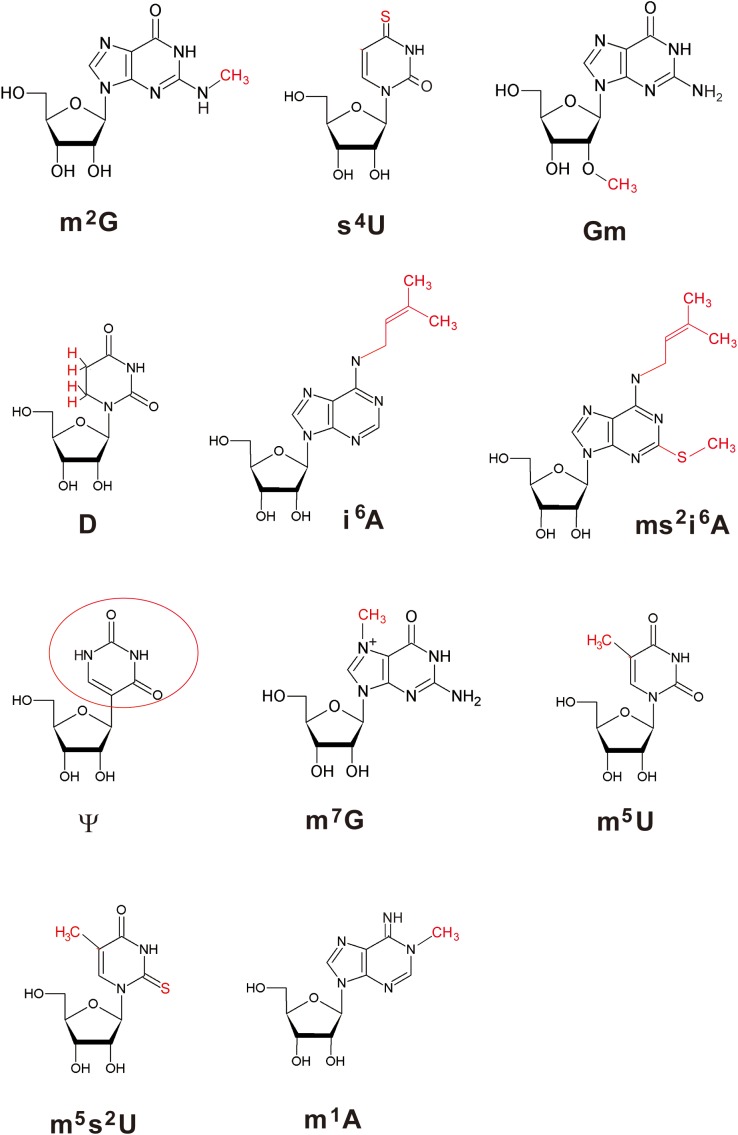
Structures of modified nucleosides in *T. thermophilus* tRNA^Phe^. The modifications are highlighted in red. Because ψ is synthesized by isomerization of uridine, the base is enclosed by a red circle.

Figure 1 shows the sequence of tRNA^Phe^ from *T. thermophilus* (Grawunder et al., 1992; Tomikawa et al., 2010). Eleven kinds of modified nucleosides indicated in red in Figure 1 have been found at ten positions in this tRNA (Figure 2); the abbreviations of modified nucleosides, related tRNA modification enzymes, and references (Watanabe et al., 1974; Caillet and Droogmans, 1988; Kammen et al., 1988; Nurse et al., 1995; Persson et al., 1997; Mueller et al., 1998; Esberg et al., 1999; Kambampati and Lauhon, 1999; Bishop et al., 2002; Hori et al., 2002; De Bie et al., 2003; Droogmans et al., 2003; Pierrel et al., 2004; Urbonavicius et al., 2005; Shigi et al., 2006a, 2016; Tomikawa et al., 2010; Ishida et al., 2011; Roovers et al., 2012; Shigi, 2012; Kusuba et al., 2015; Takuma et al., 2015; Yamagami et al., 2016; Bou-Nader et al., 2018) are summarized in Table 1. These modifications are post-transcriptionally conferred by tRNA modification enzymes, which generally act only at one position in tRNA. Thus, specific tRNA modification enzymes exist for the specific positions in specific tRNA species, even though they may synthesize the same type of modified nucleoside; for example, 2’-*O*-methylcytidine at position 32 (Cm32) in *E. coli* tRNA^Met^ is conferred by TrmJ (Purta et al., 2006), whereas Cm34 in tRNA^Leu^ is synthesized by TrmL (Benítez-Páez et al., 2010). Moreover, in several cases, multiple tRNA modification enzymes and related proteins are required for synthesis of one modified nucleoside; for example, the m^5^s^2^U54 modification in *T. thermophilus* tRNA^Phe^ requires methylation by TrmFO (Urbonavicius et al., 2005) and thiolation by TtuA, TtuB, TtuC, TtuD, and IscS (Shigi et al., 2006a, 2008, 2016; Shigi, 2012) (Table 1). In addition, the modifications in the anticodon-loop often require multiple enzymes and many substrates. For example, the synthesis of 5-methylaminomethyl-2-thiouridine at position 34 (mnm^5^s^2^U34) in *E. coli* tRNAs requires ten proteins (mnmA, mnmC, mnmE, mnmG, TusA, TusB, TusC, TusD, TusE, and IscS) and eight substrates (S-adenosyl-L-methionine, NH_4_^+^, ATP, GTP, 5, 10-methylenetetrahyrdofolate, NADH, glycine, and cysteine) (Armengod et al., 2014). Therefore, living organisms need numerous tRNA modification enzymes and related proteins beyond the multitude of modified nucleosides in tRNA.

**Table 1:** Modified nucleosides in tRNA^Phe^ from *T. thermophiles.*

Abbreviation and position	Modified nucleoside	Modification enzymes	Reference
m^2^G6	*N*^2^-methylguanosine	TrmN	Roovers et al., 2012
s^4^U8	4-thiouridine	ThiI	Mueller et al., 1998; Kambampati and Lauhon, 1999
Gm18	2′-*O*-methylguanosine	TrmH	Persson et al., 1997; Hori et al., 2002
D20	dihydrouridine	DusA	Bishop et al., 2002; Kusuba et al., 2015; Bou-Nader et al., 2018
i^6^A37	*N*^6^-isopentenyladenosine	MiaA	Caillet and Droogmans, 1988
ms^2^i^6^A37	2-methylthio- *N*^6^-isopentenyladenosine	MiaA and MiaB	Esberg et al., 1999; Pierrel et al., 2004
ψ39	pseudouridine	TruA	Kammen et al., 1988
m^7^G46	7-methylguanosine	TrmB	De Bie et al., 2003; Tomikawa et al., 2010
m^5^U54	5-methyluridine	TrmFO	Urbonavicius et al., 2005; Yamagami et al., 2016
m^5^s^2^U54	5-methyl-2-thiouridine	TrmFO, TtuA, TtuB, TtuC, TtuD and IscS	Watanabe et al., 1974; Shigi et al., 2006a, 2016; Shigi, 2012
ψ55	pseudouridine	TruB	Nurse et al., 1995; Ishida et al., 2011
m^1^A58	*N*^1^-methyladenosine	TrmI	Droogmans et al., 2003; Takuma et al., 2015


Given that *T. thermophilus* grows at high temperatures, its biomolecules including tRNA and tRNA modification enzymes show extreme heat-resistance. As a result, modified nucleosides in tRNA from *T. thermophilus* have been studied from the view-point of stabilization of tRNA structure at high temperatures. In this review, I focus on the thermal adaptation system of tRNA modifications in *T. thermophilus*, which is regulated by many factors.

## The m^5^s^2^U54 Modification in *T. thermophilus* tRNA Is Essential for Protein Synthesis at High Temperatures

### Discovery of m^5^s^2^U54 in tRNA From *T. thermophilus*

The m^5^s^2^U modification is a typical thermophile-specific modified nucleoside in tRNA (Hori et al., 2018). It was originally identified in RNase T_1_-digested RNA fragments derived from a tRNA mixture from *T. thermophilus* and sequence of the corresponding fragment strongly suggested that a portion of m^5^U at position 54 is replaced by m^5^s^2^U (Watanabe et al., 1974). Subsequently, it was found that this modified nucleoside was increased according to increasing in temperature of the cultures and melting temperature of tRNA was risen with increasing in the extent of m^5^s^2^U in tRNA (Watanabe et al., 1976). The presence of m^5^s^2^U54 was first confirmed in tRNA^Met^_f_1 and tRNA^Met^_f_2 (Watanabe et al., 1979), and then tRNA containing m^5^s^2^U54 was separated from tRNA containing m^5^U54 (Watanabe et al., 1983). The m^5^s^2^U54 modification has been identified in all *T. thermophilus* tRNA species sequenced so far [tRNA^Ile^1 (Horie et al., 1985), tRNA^Asp^ (Keith et al., 1993) and tRNA^Phe^ (Grawunder et al., 1992; Tomikawa et al., 2010)].

### Structural Effect of m^5^s^2^U54 on tRNA

The m^5^s^2^U54 modification forms a reverse-Hoogsteen base pair with A58 (or m^1^A58), and this base pair stacks with the G53-C61 base pair in the T-stem (Yokoyama et al., 1987). The hydrophobic interaction between the m^5^s^2^U54-A58 (or m^1^A58) and G53-C61 base pairs stabilizes the tertiary G18-ψ55 and G19-C56 base pairs between the T- and D-arms. Therefore, m^5^s^2^U54 contributes to stabilization of the L-shaped tRNA structure, and the melting temperature of tRNA is increased more than 3°C by the presence of m^5^s^2^U54 (Watanabe et al., 1976; Davanloo et al., 1979). Furthermore, the melting temperature of a tRNA mixture is maintained above 85°C by the 2-thiomodification in m^5^s^2^U54 (Shigi et al., 2006b), enabling *T. thermophilus* to grow at temperatures of 50–83°C.

### Temperature-Dependent Regulation of m^5^s^2^U54 Content in tRNA^Phe^ and Protein Synthesis

**FIGURE 3:**
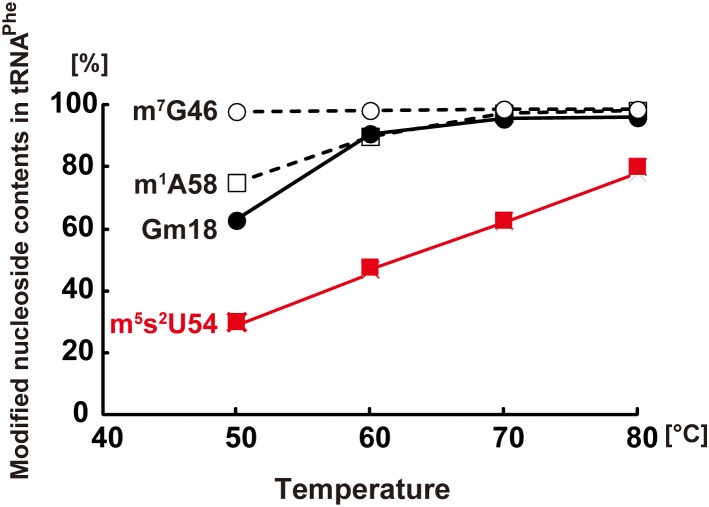
Extents of m^7^G46, m^1^A58, Gm18, and m^5^s^2^U54 modifications in tRNA^Phe^ from *T. thermophilus*. The extent of m^7^G46, m^1^A58, and Gm18 modifications was measured by a methylation assay with TrmB, TrmI, and TrmH, respectively. The extent of m^5^s^2^U54 modification was estimated from the peak areas of m^5^U54 and m^5^s^2^U54 on HPLC analysis. This figure is prepared from Figure 3 in a chapter “Regulation of Protein Synthesis via the Network Between Modified Nucleotides in tRNA and tRNA Modification Enzymes in *T. thermophilus*, a Thermophilic Eubacterium” of a book “Modified Nucleic Acids in Biology and Medicine,” Springer Nature 2016 with permission (4517441319562) from the publisher.

Figure 3, which is based on a combination of previous experimental results, shows the percentage of different modified nucleosides (m^5^s^2^U54, Gm18, m^7^G46, and m^1^A58) in tRNA^Phe^, purified from *T. thermophilus* cells cultured at 50, 60, 70, and 80°C (Tomikawa et al., 2010; Ishida et al., 2011; Yamagami et al., 2016). As shown in Figure 3, the extent of m^5^s^2^U54 in tRNA^Phe^ increases with increasing culture temperatures. The balance between m^5^s^2^U54 and m^5^U54 regulates the rigidity (flexibility) of tRNA at a wide range of temperatures (50–83°C).

The presence of m^5^s^2^U54 in tRNA is required for efficient protein synthesis at high temperatures. A previous study measured the activity of Poly (U)-dependent poly-phenylalanine synthesis at several temperatures using various fractions (tRNA^Phe^, ribosome, and supernatant of 100,000 × *g* centrifugation fraction) prepared from *T. thermophilus* cells cultured at 50 and 80°C (Yokoyama et al., 1987). Poly-phenylalanine was effectively synthesized at 80°C only when tRNA^Phe^ from cells cultured at 80°C was used. As shown in Figure 3, the proportion of m^5^s^2^U54 in tRNA^Phe^ from cells cultured at 80°C is higher than that in tRNA^Phe^ from cells cultured at 50°C. The melting temperature of a tRNA mixture from cells cultured at 70°C is 79.7°C in the presence of 50 mM Tris–HCl (pH7.5), 5 mM MgCl_2_ and 100 mM NaCl (Tomikawa et al., 2010); thus, the structure of tRNA^Phe^ from cells cultured at 50°C seems to be looser than that from cells cultured at 80°C. Indeed, the gene disruption strain of *ttuA* encoding a component of sulfur-transfer complex for the m^5^s^2^U54 formation, results in a growth defect of the *T. thermophilus* strain at 80°C (Shigi et al., 2006a). Thus, the m^5^s^2^U54 modification is essential for effective protein synthesis in *T. thermophilus* at high temperatures.

### m^5^s^2^U54 in tRNA From Other Thermophiles

In addition to *T. thermophilus*, m^5^s^2^U has been identified among the modified nucleosides in unfractionated tRNA from *Thermotoga maritima* (Edmonds et al., 1991) and at position 54 in tRNA^Cys^ from *Aquifex aeolicus* (Awai et al., 2009). Furthermore, the m^5^s^2^U nucleoside has been identified among the modified nucleosides in unfractionated tRNA from some hyper-thermophilic archaea such as *Thermococcus* species (Edmonds et al., 1991) and *Pyrococcus furiosus* (Kowalak et al., 1994). Therefore, it is considered that these hyper-thermophiles also possess the m^5^s^2^U54 modification in tRNA.

### Biosynthetic Pathway of m^5^s^2^U54 in Eubacteria and Archaea

Biosynthesis of m^5^s^2^U54 is accomplished in two steps, methylation of the C5 atom and the 2-thiolation. Although these steps occur independently at U54 in tRNA (Shigi et al., 2006a; Yamagami et al., 2016), the m^5^U54 modification in tRNA is almost fully formed in living cells even when *T. thermophilus* is cultured under nutrient-poor conditions (Yamagami et al., 2018). To date, therefore, an s^2^U54 modification has not been observed in tRNA from the *T. thermophilus* wild-type strain.

Formation of m^5^U54 is catalyzed by different tRNA methyltransferases in bacteria and archaea. A folate/FAD-dependent tRNA methyltransferase (TrmFO) catalyzes the methylation using 5, 10-methylenetetrafolate as a methyl donor in bacteria (Urbonavicius et al., 2005; Nishimasu et al., 2009; Yamagami et al., 2012; Hamdane et al., 2016), whereas an S-adenosyl-L-methionine (AdoMet)-dependent tRNA methyltransferase [RumA (or TrmA)-like enzyme] works in archaea (Urbonavicius et al., 2008). Notably, *E. coli* TrmA is an AdoMet-dependent tRNA (m^5^U54) methyltransferase (Ny and Björk, 1980), whereas *E. coli* RumA is an AdoMet-dependent 23S rRNA (m^5^U1939) methyltransferase (Agarwalla et al., 2002; Madsen et al., 2003). Although these RNA m^5^U methyltransferases belong to the same cluster of orthologous proteins (group COG2265) (Urbonavicius et al., 2008), archaeal tRNA (m^5^U54) methyltransferases structurally resemble RumA rather than TrmA (Walbott et al., 2008). Therefore, archaeal tRNA (m^5^U54) methyltransferases for m^5^s^2^U54 formation might have evolved from a RumA-type rRNA methyltransferase.

The 2-thiolation in m^5^s^2^U54 of *T. thermophilus* is conferred by multiple proteins, namely TtuA, TtuB, TtuC, TtuD and IscS (or SufS) (Shigi et al., 2006a, 2008, 2016; Shigi, 2012; Chen et al., 2017). In addition, the mechanism of the sulfur-transfer reaction carried out by TtuA from *T. maritima* has been recently proposed based on crystal structures of the enzyme (Arragain et al., 2017). The protein factors involved in 2-thiolation of m^5^s^2^U54 in archaea have not been confirmed experimentally.

## tRNA Modification Enzymes Recognizes the Local Structure(s) in tRNA

**FIGURE 4:**
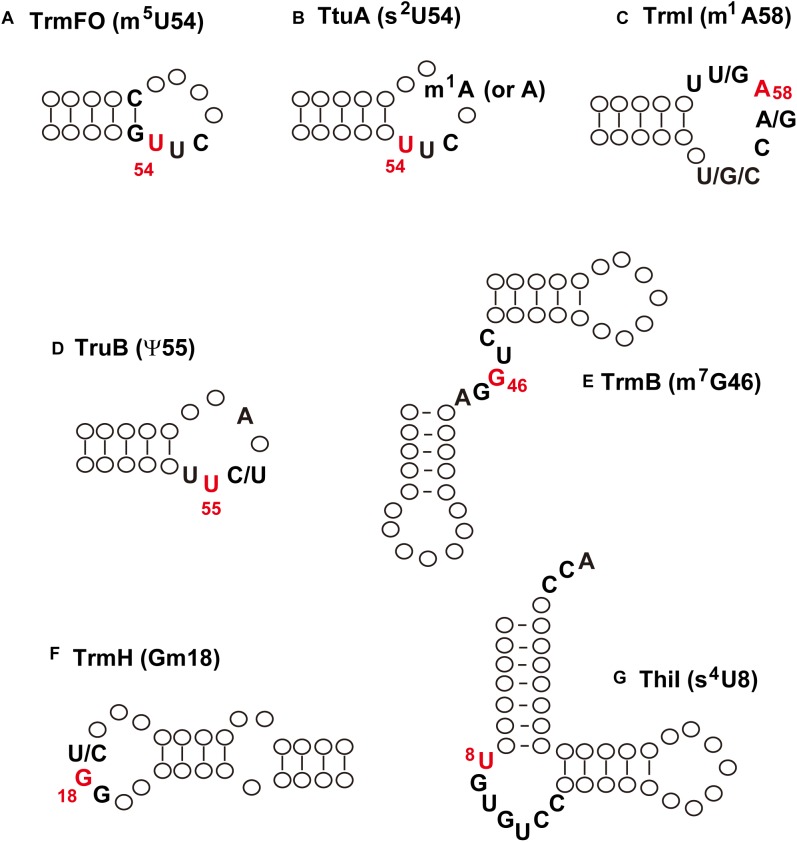
Minimum substrate RNA or positive determinants for tRNA modification enzymes. **(A)** TrmFO; **(B)**, TtuA; **(C)**, TrmI; **(D)**, TruB; **(E)**, TrmB; **(F)** TrmH; **(G)** ThiI. The modification is indicated in parentheses; the modification site is colored in red. See the main text for details.

At the beginning of this century, the mechanisms of regulating the extent of modified nucleosides in tRNA from *T. thermophilus* were unknown. The transcriptional and/or translational regulations of amounts of tRNA modification enzyme(s) were assumed at the start of our studies, however, we noticed that this regulation might be explainable by the substrate tRNA recognition mechanisms of the tRNA modification enzymes. In general, tRNA modification enzymes recognize the local structure in tRNA. Figure 4 shows the minimum substrate or positive determinants for different tRNA modification enzymes, which I describe in more detail below.

### m^5^U54 Formation by TrmFO

TrmFO can methylate a micro-helix RNA, which mimics the T-arm structure (Yamagami et al., 2012; Figure 4A). The positive determinants for TrmFO are the stem-loop structure, G53-C61 base pair, and U54U55C56 sequence. Therefore, the substrate RNA recognition mechanism of TrmFO is very simple. This simple structure is also observed in the anticodon-loop of tRNA^Pro^ from *T. thermophilus*; however, A38 in the anticodon-loop prevents incorrect methylation by TrmFO (Yamagami et al., 2012). In some cases, therefore, there are negative determinants for tRNA modification enzymes.

### s^2^U54 Formation by TtuA

In the case of TtuA (Figure 4B), only the modification patterns of tRNA^Asp^ mutants expressed in *T. thermophilus* cells have been analyzed (Shigi et al., 2002); therefore, it is unknown whether TtuA can act on a micro-helix RNA. Nevertheless, it is clear that the positive determinants for the sulfur-transfer reaction of TtuA are also very simple. More recently, it was shown that the presence of m^1^A58 accelerates the velocity of sulfur-transfer (Shigi et al., 2006b). Thus, the sulfur-transfer reaction carried out by TtuA is regulated by another modification (m^1^A58).

### m^1^A58 Formation by TrmI

The tRNA m^1^A58 methyltransferase TrmI can methylate a micro-helix RNA very slowly (Takuma et al., 2015; Figure 4C). The presence of an aminoacyl-stem or variable region accelerates the rate of methylation of truncated tRNA by TrmI. Furthermore, the presence of an m^7^G46 modification also accelerates the rate of methylation by TrmI (Tomikawa et al., 2010). Thus, the extent of m^1^A58 modification in tRNA is also controlled by the presence of another modification (m^7^G46).

Among *T. thermophilus* tRNAs, tRNA^Thr^_GGU_ exceptionally possesses C60 instead of U60, which is one of the positive determinants for TrmI. The proportion of m^1^A58 and m^5^s^2^U54 in tRNA^Thr^_GGU_ (Kazayama et al., 2015) is lower than that in tRNA^Phe^ (Takuma et al., 2015), indicating that the extent of m^1^A58 modification has an effect on the extent of m^5^s^2^U54 modification, which is consistent with the observation of Shigi et al. (2006b). Thus, the degree of m^1^A58 modification in tRNAs differs according to the sequence of each tRNA. The sequence of tRNA^Thr^_GGU_ seems to be disadvantageous for survival of *T. thermophilus* at high temperatures, however, the physiological reason for the presence of C60 in tRNA^Thr^_GGU_ is unknown.

### ψ55 Formation by TruB

*Escherichia coli* TruB can modify a micro-helix RNA, which mimics the T-arm structure (Gu et al., 1998). U54, U55, pyrimidine56 and A58 in the T-loop are important for the full activity of *E. coli* TruB (Figure 4D; Gu et al., 1998). The crystal structure of a complex of TruB and micro-helix RNA showed that other nucleotides in the T-loop contact with TruB (Hoang and Ferré-D’Amaré, 2001; Pan et al., 2003). The ribose-phosphate backbone in the T-arm structure, which is formed by the U54-A58 reverse Hoogsteen base pair, is important for the reaction by *E. coli* TruB. Although the substrate tRNA recognition mechanism of *T. thermophilus* TruB has not been confirmed experimentally, the high conservation of amino acid sequences between *E. coli* and *T. thermophilus* TruB proteins (Ishida et al., 2011) strongly suggests that these enzymes possess a common mechanism of tRNA recognition. Formation of ψ55 in *T. thermophilus* tRNA^Phe^ transcript by TruB is very rapid at 55°C (Ishida et al., 2011), suggesting that *T. thermophilus* TruB does not require other modifications in tRNA in order to function.

### m^7^G46 Formation by TrmB

*Aquifex aeolicus* TrmB can methylate a truncated tRNA (Figure 4E): its methylation speed for truncated RNA is comparable to that for the full-length tRNA transcript (Okamoto et al., 2004). In the truncated tRNA, the T-arm-like structure and five nucleosides corresponding to variable region are essential for methylation by TrmB. Thermophilic TrmB (*A. aeolicus* and *T. thermophilus* TrmB) share considerable amino acid sequence homology and exceptionally possess a long C-terminal region (Okamoto et al., 2004), which is involved in binding to AdoMet (Tomikawa et al., 2018). Therefore, the substrate tRNA recognition mechanism of thermophilic TrmB may be different from that of mesophilic TrmB (De Bie et al., 2003; Purta et al., 2005; Zegers et al., 2006; Zhou et al., 2009; Tomikawa, 2018).

### Gm18 Formation by TrmH

Figure 4F shows the minimum substrate RNA of *A. aeolicus* TrmH (Hori et al., 2003). There are some differences in substrate tRNA recognition mechanism between *A. aeolicus* TrmH and *T. thermophilus* TrmH. For example, *A. aeolicus* TrmH cannot methylate tRNAs with A17 (Hori et al., 2003), but *T. thermophilus* TrmH methylates tRNA^Ser^, which contains A17 (Hori et al., 1998). Furthermore, the size and sequence of the D-loop have effects on methylation by *A. aeolicus* TrmH (Hori et al., 2003), however, *T. thermophilus* TrmH methylates mutant tRNAs irrespective of these D-loop features (Ochi et al., 2010). In short, *T. thermophilus* TrmH has been found to methylate all tRNAs tested so far (Hori et al., 1998). *T. thermophilus* TrmH can methylate a 5’-half fragment of tRNA (Matsumoto et al., 1990) but not a micro-helix that mimics the D-arm (Hirao et al., 1989). Therefore, *T. thermophilus* TrmH may recognize the bulge structure like *A. aeolicus* TrmH. This idea is consistent with observations that the cross-linking or chemical modification of s^4^U8 in substrate tRNA causes a decrease in methylation speed by TrmH (Hori et al., 1989). Full activity of TrmH requires the L-shaped structure formed by conserved nucleosides in tRNA (Hori et al., 1998). Furthermore, the speed of methylation by *T. thermophilus* TrmH for yeast tRNA^Phe^ transcript is slower than that for native yeast tRNA^Phe^ at 65°C, showing that other modified nucleosides have a positive effect on the methylation by TrmH (Hori et al., 1998). Indeed, the presence of m^7^G46 in tRNA^Phe^ transcript accelerates methylation speed by TrmH (Tomikawa et al., 2010).

### s^4^U8 Formation by ThiI

*Escherichia coli* and *T. maritima* ThiI (tRNA 4-thiouridine synthetase) can modify several truncated tRNAs that mimic the aminoacyl-stem and T-arm (Figure 4G; Lauhon et al., 2004; Neumann et al., 2014). Although the substrate tRNA recognition mechanism of *T. thermophilus* ThiI has not been investigated, it is likely that *T. thermophilus* ThiI also recognizes the local structure in tRNA. The THUMP domain in ThiI recognizes the CCA terminus of substrate RNA (Neumann et al., 2014). This feature has been identified in another tRNA modification enzyme, archaeal Trm11, which also possesses a THUMP domain (Hirata et al., 2016). Furthermore, given that TrmN and archaeal Trm14 have a THUMP domain (Menezes et al., 2011; Fislage et al., 2012; Roovers et al., 2012), these enzymes may recognize the CCA terminus in tRNA.

### D20 and D20a Formations by DusA

In *T. thermophilus*, single Dus family protein, DusA synthesizes all D modifications (D20 and D20a) in tRNA (Kusuba et al., 2015); in *E. coli*, by contrast, three Dus family proteins share the modification sites in tRNA (Bishop et al., 2002; Bou-Nader et al., 2018). Among the *T. thermophilus* tRNA modification enzymes that act on the three-dimensional core in tRNA, DusA exceptionally recognizes the interaction between the T-arm and D-arm (Yu et al., 2011). For the reaction of DusA at high temperatures, therefore, stabilization of the L-shaped tRNA structure by other modified nucleosides is essential (Kusuba et al., 2015). Thus, D20 and D20a seem to be relatively late modifications in *T. thermophilus* tRNA.

## Initial Binding and Induced-Fit Steps in Complex Formation Between tRNA Modification Enzymes and Substrate tRNA

As shown in Figure 4, many tRNA modification enzymes recognize the local structure in tRNA, while the interaction between the T-arm and D-arm in tRNA is not required for their activity. Indeed, in the case of TrmI, the methylation speed is faster for a mutant tRNA transcript, in which the interaction between the T-arm and D-arm is disrupted, than for the wild-type tRNA transcript (Takuma et al., 2015).

In many cases, the target modification site is embedded in the L-shaped tRNA structure. For example, G18 and U55 form a tertiary base pair and the uracil base in U55 is not localized at the surface of tRNA. Therefore, disruption of L-shaped tRNA structure is necessary for the reaction of TruB. Furthermore, to fit into the catalytic pocket of TruB, the uracil base must be flipped. These observations suggest that the reaction of tRNA modification enzyme comprises at least two steps, initial binding to the L-shaped tRNA, followed by a structural change (induced-fit) process in which the L-shaped tRNA structure is disrupted.

**FIGURE 5:**
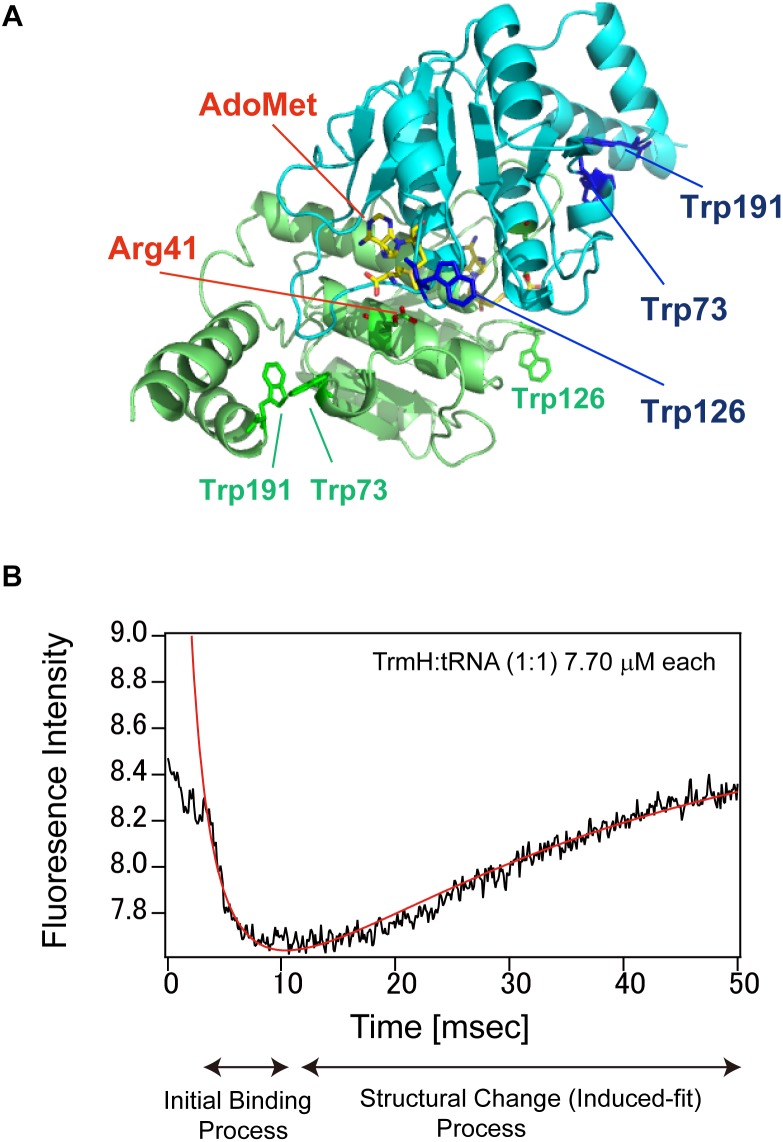
TrmH and the pre-steady state analysis of complex formation between TrmH and tRNA. **(A)** Dimer structure of TrmH. AdoMet, Arg41 and tryptophan residues are highlighted by stick-models. The two subunits of TrmH are distinguished by green and blue coloring. Trp126 residue is located near AdoMet in the blue subunit and Arg41 in the green subunit. **(B)** Stopped-flow fluorescence measurement of TrmH–tRNA complex formation. See main text for details.

These steps have been monitored for complex formation between *T. thermophilus* TrmH and tRNA^Phe^ transcript by using a stopped-flow fluorescence measurement system (Figure 5; Ochi et al., 2010, 2013). *T. thermophilus* TrmH is a member of the SpoU-TrmD (so-called SPOUT) methyltransferase superfamily (Anantharaman et al., 2002; Nureki et al., 2004; Hori, 2017), and site-directed mutagenesis studies (Nureki et al., 2004; Watanabe et al., 2005) suggest that arginine at position 41 (Arg41) in TrmH acts as the catalytic center. As shown in Figure 5A, TrmH is a dimeric enzyme with three tryptophan residues (Trp73, Trp126, and Trp191) in each subunit. The fluorescence intensity at 320 nm derived from these tryptophan residues was measured during complex formation between TrmH and tRNA. Figure 5B shows the one result obtained when 7.70 μM TrmH–AdoMet complex and 7.70 μmM tRNA^Phe^ transcript were mixed by the stopped-flow system at 25°C (Ochi et al., 2010). A very fast decrease in fluorescence was observed in the initial 10 ms, followed by relatively slow increase in fluorescence from 10 to 50 ms. In general, a decrease of tryptophan fluorescence intensity suggests an increase in accessibility of the residue to solvent water. The obtained data could be fitted to an equation, which showed that the reaction was bi-molecular binding reaction. In the case of TrmH, therefore, the initial decrease of fluorescence suggests that a tryptophan residue(s) is moved to the solvent during the initial binding process. Subsequently, this residue was confirmed as Trp126 by measurements on TrmH mutant proteins (Ochi et al., 2013). The slow increase in fluorescence from 10 to 50 ms reflects the structural change (induced-fit) process; detailed measurements on several concentrations of TrmH and tRNA indicate that the slow increase in fluorescence is fitted to a combination of unimolecular (first-order) reactions. The methylation was not completed within 50 ms, showing that the slow increase in fluorescence was not caused by dissociation of tRNA from the enzyme after the reaction. In the structural change process, Trp126 is moved to a hydrophilic environment. During the induced fit process, disruption of L-shaped tRNA structure, recognition of 6-oxygen in G18, and introduction of ribose into the catalytic pocket are occurred (Ochi et al., 2010).

The folding of tRNA and rigidity (flexibility) of the local structure in tRNA affect the speed of the initial binding and induced-fit processes. Thus, other modifications in tRNA, temperature, and RNA stabilization factors such as Mg^2+^ ions and polyamines are all likely to influence on the initial binding and induced-fit processes.

## A Network Between Modified Nucleosides in tRNA and tRNA Modification Enzymes Controls the Flexibility (Rigidity) of tRNA in *T. thermophilus* at a Wide Range of Temperatures

**FIGURE 6:**
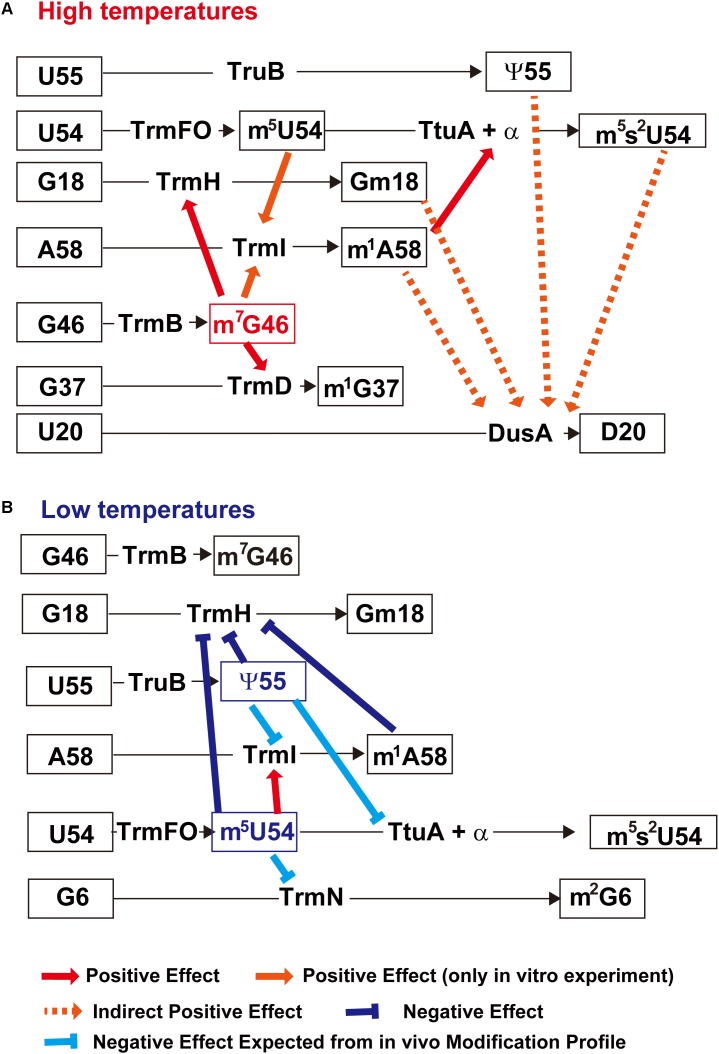
Network between modified nucleosides in tRNA and tRNA modification enzymes in *T. thermophilus.* **(A)** Network at high temperatures (>75°C). The m^7^G46 modification (highlighted in red) is a key factor in this network. Its presence accelerates the speed of other tRNA modification enzymes such as TrmH, TrmD, and TrmI. In addition, the presence of m^5^U54 also increases the methylation speed of TrmI. The increase in m^1^A58 due to accelerated TrmI activity further increases the speed of sulfur-transfer by TtuA and that of related proteins, and results in an increased percentage of m^5^s^2^U54. The introduced modifications coordinately stabilize the L-shaped tRNA structure. **(B)** Network at low temperatures (<55°C). In this network, the ψ55 modification stabilizes the local structure in tRNA and slows down the speed of tRNA modification enzymes. The m^5^U54 modification plays a role in maintaining the balance of modifications at the elbow region in tRNA. This figure is prepared from Figure 4 in a chapter “Regulation of Protein Synthesis via the Network Between Modified Nucleotides in tRNA and tRNA Modification Enzymes in *T. thermophilus*, a Thermophilic Eubacterium” of a book “Modified Nucleic Acids in Biology and Medicine” Springer Nature 2016 with permission (4517441319562) from the publisher.

A method for preparing gene disruptant strain of *T. thermophilus* was developed at the beginning of this century (Hoseki et al., 1999; Hashimoto et al., 2001). This gene-disruption system, coupled with biochemical studies, has been used to elucidate the regulatory network between modified nucleosides in tRNA and tRNA modification enzymes in *T. thermophilus* cells. Figure 6 summarizes the network between modified nucleosides in tRNA and tRNA modification enzymes. Although each tRNA modification enzymes can act on unmodified tRNA transcript (or truncated tRNA transcript as shown in Figure 4), the presence of modified nucleosides often accelerates (or slows down) the speed of modification by other tRNA modification enzymes depending on the environmental temperatures.

*T. thermophilus* lives in hot springs. The temperature of hot spring water can change for several reasons including an influx of river water, snowfall, and eruption of hot water. Therefore, the ability of protein synthesis to adapt to temperature changes via the flexibility (rigidity) of tRNA is very important for survival of *T. thermophilus*. One of advantages of the network is that it does not require protein synthesis. As a result, it can respond rapidly. Furthermore, the network may be a survival strategy of eubacteria, which have a limited genome size.

### Network at High Temperatures (>75°C)

At high temperatures (>75°C), m^7^G46 modification by TrmB is one of the key modifications in the network (Figure 6A). It has been shown that the *trmB* gene deletion strain does not grow at 80°C and has several hypo-modifications in tRNA (Tomikawa et al., 2010). When the culture temperature is shifted from 70 to 80°C, tRNA^Phe^ and tRNA^Lys^ are degraded and protein synthesis is impaired in this strain. Particular, heat shock proteins are not synthesized efficiently in the *trmB* gene deletion strain. Thus, the m^7^G46 modification is essential for survival of *T. thermophilus* at high temperatures.

The positive effects of m^7^G46 on TrmH, TrmD, and TrmI activity have been confirmed by *in vitro* experiments. Because the m^1^G37 modification conferred by TrmD is not present in *T. thermophilus* tRNA^Phe^, yeast tRNA^Phe^ transcript was used in these experiments (Tomikawa et al., 2010). TrmH can methylate a 5’-half fragment of tRNA (Matsumoto et al., 1990), but its full activity requires the three-dimensional core structure of tRNA (Hori et al., 1998). m^7^G46 forms a tertiary base pair with the C13-G22 base pair in the D-arm; thus, this tertiary base pair seems to have a positive effect on TrmH activity. TrmD can act on a truncated tRNA (Redlak et al., 1997) and micro-helix RNA (Takeda et al., 2006), but foot-printing analyses have shown that the D-arm and variable region are protected in addition to the anticodon-arm (Gabryszuk and Holmes, 1997). Furthermore, the crystal structure of the TrmD–tRNA complex revealed that the C-terminal domain of TrmD makes contacts with the D-arm in tRNA (Ito et al., 2015). Therefore, the positive effect of m^7^G46 on TrmD activity can be explained by stabilization of the D-arm via the formation of an m^7^G46-C13-G22 tertiary base pair.

In the case of TrmI, the presence of aminoacyl-stem or variable region increases the methyl-group acceptance activity of a truncated tRNA transcript (Takuma et al., 2015). Although there is no docking model of TrmI with tRNA, positively-charged grooves, which are present on the surface of TrmI (Barraud et al., 2008), may capture the aminoacyl-stem and variable region in tRNA.

The positive effect of m^5^U54 on TrmI activity was also confirmed *in vitro* (Yamagami et al., 2012). However, the tRNA fraction from a *trmFO* gene deletion strain cultured at 70°C contained the same amount of m^1^A nucleoside as that from the wild-type strain (Yamagami et al., 2016). Therefore, there seems to be a sufficient amount of TrmI to maintain the extent of m^1^A58 in tRNA in the *T. thermophilus trmFO* gene deletion strain. The positive effect of m^5^U54 on the TrmI activity can be explained by stabilizing effect of the m^5^U54-A58 reverse Hoogsteen base pair.

As described in the Section “tRNA Modification Enzymes Recognizes the Local Structure(s) in tRNA”, the m^1^A58 modification conferred by TrmI is a positive determinant for the sulfur-transfer system (TtuA and related proteins) (Shigi et al., 2006b). Therefore, TrmI is essential for survival of *T. thermophilus* at high temperatures (Droogmans et al., 2003). Furthermore, the m^5^s^2^U54 modification is essential for stabilizing the tRNA structure, as described in the Section “The m^5^s^2^U54 Modification in *T. thermophilus* tRNA is Essential for Protein Synthesis at High Temperatures.” As a result, the sulfur-transfer system for s^2^U54 formation is also essential for the survival of *T. thermophilus* at high temperatures (Shigi et al., 2006a). In contrast, TrmFO is not essential and the *trmFO* gene deletion strain can grow at 80°C (Yamagami et al., 2016). Collectively, these modified nucleosides in tRNA coordinately stabilize the tRNA structure at high temperatures.

Given that the formation of D20 by DusA requires the interaction between D-arm and T-arm (Yu et al., 2011), stabilization of the L-shaped tRNA structure is essential for D20 formation at high temperatures (Kusuba et al., 2015). Therefore, several modifications, including m^5^s^2^U54, m^1^A58, Gm18 and ψ55, seem to be required for sufficient activity of DusA at high temperatures.

Among the modified nucleosides in *T. thermophilus* tRNA^Phe^, m^2^G6 by TrmN, s^4^U8 by ThiI, i^6^A37 and ms^2^i^6^A37 by MiaA and MiaB, and ψ39 by TruA have not been investigated as yet. However, it is possible that some of them may affect the stability of tRNA in *T. thermophilus* at high temperatures. For example, it has been recently reported that the melting temperature of tRNA from *E. coli thiI* gene disruptant strain is lower than that from the wild-type strain (Nomura et al., 2016). Therefore, s^4^U8 may contribute to stabilization of the structure of tRNA. In the case of anticodon-loop modifications (ψ38, i^6^A37, and ms^2^i^6^A37), their deletion may severely impair protein synthesis because they are important for the structure of anticodon-loop and function directly in protein synthesis (Spenkuch et al., 2014; Grosjean and Westhof, 2016; Schweizer et al., 2017). Furthermore, i^6^A37 is required for the 2’-*O*-methylation conferred by TrmL at position 34 in *E. coli* tRNA^Leu^ (Benítez-Páez et al., 2010; Zhou et al., 2015). In the case of *T. thermophilus* tRNA^Leu^, therefore, TrmL is probably included in the network.

### Network at Low Temperatures (<55°C)

At low temperatures (<55°C), the ψ55 modification conferred by TruB works as a key factor in the network (Figure 6B). In the *truB* gene deletion strain, excess amounts of Gm18, m^1^A58 and m^5^s^2^U54 are introduced into tRNAs at low temperatures and the melting temperature of tRNA mixture is increased by more than 8°C (Ishida et al., 2011). This excess rigidity of tRNA results in a disorder of protein synthesis, and cold shock proteins are not synthesized efficiently in the *truB* gene deletion strain. Therefore, ψ55 in tRNA is required for survival of *T. thermophilus* at low temperatures. The m^5^U54 modification aids ψ55 in maintaining the balance of other modifications in tRNA (Yamagami et al., 2016). The ψ55 modification stabilizes the structure of elbow region in tRNA and slows down the formation speed of other modifications around ψ55 (Gm18, m^1^A58 and m^5^s^2^U54) (Ishida et al., 2011). The positive effect of m^5^U54 on m^1^A58 modification was confirmed both by the *in vivo* methylation profile and by *in vitro* experiments (Yamagami et al., 2016) and is probably due to the stabilization of the m^5^U54-A58 reverse Hoogsteen base pair.

## What Stabilizes the Structure of Unmodified Precursor tRNA in *T. thermophilus* at 80°C?

Unmodified tRNA transcript cannot maintain its L-shaped tRNA structure at high temperatures. Primary transcript (precursor tRNA), which is synthesized by RNA polymerase, is unmodified. Therefore, even though several tRNA modification enzymes from *T. thermophilus* can act on unmodified tRNA transcript, their activities cannot be measured at 80°C due to the disrupted structure of substrate RNA. For example, *T. thermophilus* TrmH methylates tRNA effectively only at temperatures below the melting temperature (Matsumoto et al., 1987). These observations raise an important question, what stabilizes the structure of unmodified precursor tRNA in *T. thermophilus* at 80°C? If there were no stabilization factors in living cells, tRNA modification enzymes from *T. thermophilus* would not be able to act on precursor tRNA at 80°C.

### Unique Polyamines in *T. thermophilus* and Their Interaction With tRNA

**FIGURE 7:**
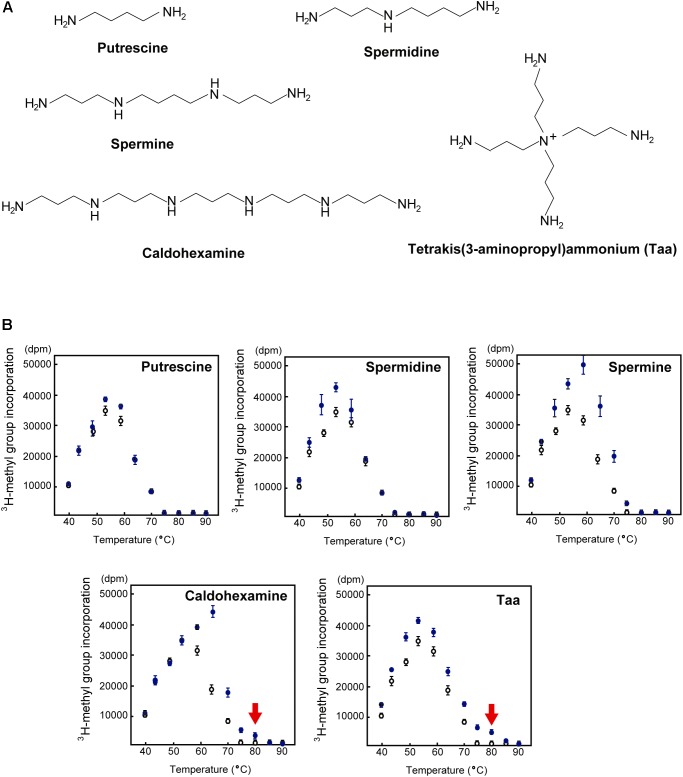
Effect of polyamines on TrmH activity. **(A)** Typical polyamines. Putrescine, spermidine, and spermine are the three standard polyamines that are found in all living organisms. Unique long and branched polyamines such as caldohexamine and tetrakis(3-aminopropyl)ammonium (Taa) exist in *T. thermophilus*. **(B)** TrmH activity was measured in the presence (blue filled circles) or absence (black open circles) of polyamines at 40–90°C. Only in the presence of appropriate concentrations of caldohexamine (1.5 mM) or Taa (0.25 mM), *T. thermophilus* TrmH can methylate yeast tRNA^Phe^ transcript, of which the melting temperature is 69°C, at 80°C (red arrow).

In general, living organisms produce three standard polyamines (putrescine, spermidine and spermine) (Bae et al., 2018; Igarashi and Kashiwagi, 2018). In addition to these standard polyamines, *T. thermophilus* produces at least 16 polyamine species, including long and branched polyamines (Figure 7A; Hamana et al., 1991; Oshima, 2007; Oshima et al., 2011).

Because polyamines have positive charges and hydrophilic regions, they have the potential to interact with nucleic acids and phospholipids. Indeed, there have been several studies on the interaction between polyamines and tRNA. For example, addition of polyamines shifted the melting temperature of native tRNA^Phe^ from *Saccharomyces cerevisiae* to higher temperatures in accordance with polyamine length (Terui et al., 2005). The crystal structure of the complex of yeast tRNA^Phe^ and spermine revealed that two spermine molecules bind to two sites in one tRNA^Phe^ molecule (Quigley et al., 1978). Furthermore, FT-IR analysis of tRNA in the presence of putrescine, spermidine, and spermine showed that similar to spermine, putrescine and spermidine bind to the connection region between the D-arm and anticodon-stem (Ouameur et al., 2010). Moreover, a ^13^C-NMR study reported that 14 spermidine-binding sites were present in one tRNA molecule and that three spermidine molecules stably bound to tRNA in the presence of Mg^2+^ (Frydman et al., 1990). It has also been reported that a branched polyamine (Figure 7A), tetrakis(3-aminopropyl)ammonium (Taa), slightly stimulates the activity of archaeal Trm1 and TrmI (Hayrapetyan et al., 2009), which are archaeal tRNA methyltransferases for the formation of m^2^_2_G26 (or m^2^G26) (Constantinesco et al., 1999) and m^1^A57 and m^1^A58 (Roovers et al., 2004), respectively.

### TrmH Methylates Unmodified tRNA Transcript at 80°C Only in the Presence of Long or Branched Polyamine

The effect of polyamine on the methyl-transfer speed of TrmH for yeast tRNA^Phe^ transcript has been measured at various temperatures (Figure 7B; Hori et al., 2016). All polyamines were found to increase the speed of methylation by TrmH at appropriate temperatures, although the positive effect of putrescine was relatively weak and observed only at low temperatures. As the length of polyamine increased, the optimum temperature for methyl-transfer shifted to higher temperatures, however, standard polyamines did not work at 80°C. By contrast, very weak but clear methyl-transfer activity of TrmH for unmodified tRNA^Phe^ was observed at 80°C in the presence of optimum concentration (1.5 mM) of caldohexamine, a long polyamine, (red arrow in Figure 7B). Addition of branched polyamine Taa had a stronger positive effect on TrmH activity at 80°C (red arrow in Figure 7B). Thus, long and branched polyamines can support the methylation by TrmH at 80°C.

If initial modifications are introduced into unmodified precursor tRNA, they stabilize the local structure in tRNA enabling the tRNA modification enzymes to function on the precursor tRNA. Introduction of initial modifications into tRNA probably occurs in the presence of polyamines. Among polyamines, long and branched polyamines are effective at very high temperatures.

Because TrmB, which confers the m^7^G46 modification, is a key enzyme in the network at high temperatures, the effects of polyamines on TrmB activity should be clarified. At present, however, this is not possible due to a technical problem: *T. thermophilus* TrmB, which is expressed in *E. coli* cells, is partially degraded in these cells and purification of intact TrmB is difficult.

### Long and Branched Polyamines Are Required for Maintenance of 70S Ribosome and Several tRNAs

The biosynthetic pathway from arginine to spermidine in *T. thermophilus* is different from that in eukaryotes, archaea, and other bacteria because the intermediate in *T. thermophilus* is *N*^1^-aminopropylagmatine (Ohnuma et al., 2005, 2011). *S*-Adenosyl-L-methionine decarboxylase-like protein 1 (SpeD1) is required for the biosynthesis of *N*^1^-aminopropylagmatine from arginine, and aminopropylagmatine ureohydrolase (SpeB) catalyzes the conversion of *N*^1^-aminopropylagmatine to spermidine. Because long and branched polyamines are synthesized from spermidine, the *T. thermophilus speD1* or *speB* gene deletion strain cannot produce long and branched polyamines (Nakashima et al., 2017) and cannot grow at high temperatures (>75°C) unless polyamines are added to the medium. When the *speD1* and *speB* deletion strains were cultured at 70°C in minimal medium until mid-log phase and then the culture temperature was shifted to 80°C, they could survive for 10 h. Although abnormal modifications in tRNA were expected, at least the m^5^U54, m^7^G46, Gm18 and m^1^A58 modifications were present as normal in the tRNA mixture from these strains. Given that the transcription of tRNA and the introduction of major modifications into tRNA are expected to occur mainly before the mid-log phase, it seems that these modification can be introduced into tRNA at 70°C without the presence of long and branched polyamines: the expression patterns of mRNAs in the wild-type strain can be obtained from the database (NCBI/GEO^2^) (Shinkai et al., 2007). After the temperature shift, tRNA^His^, tRNA^Tyr^ and 70S ribosome were gradually degraded in the *speD1* and *speB* deletion strains and protein synthesis was severely impaired (Nakashima et al., 2017). Thus, long and branched polyamines are required to maintain several tRNAs at high temperatures in *T. thermophilus* in addition to regulating the extent of modified nucleosides in tRNA.

### Other Regulatory Factors for tRNA Stability

RNA binding proteins, Mg^2+^ ions and K^+^ ions can stabilize tRNA structure.

In *A. aeolicus*, a hyper-thermophilic bacterium, tRNA-binding protein 111 (Trbp111) stabilizes the three-dimensional core of tRNA (Morales et al., 1999; Swairjo et al., 2000). However, Trbp111 is specific to *A. aeolicus* and not found in *T. thermophilus*. Archease is also an RNA-binding protein that changes the specificity of archaeal Trm4 (Auxilien et al., 2007) and is required for tRNA splicing (Desai et al., 2014; Popow et al., 2014). Although neither *trm4* nor a tRNA gene with an intron-coding region is not encoded in the *T. thermophilus* genome, an archease-like protein gene (TTHA1745) exists. Therefore, it is possible that an archease-like protein stabilizes tRNA structure at high temperatures in *T. thermophilus* cells.

Mg^2+^ ions are required for folding of tRNA (Lorenz et al., 2017) and are important in considering the structural effects of several modifications in tRNA (Yue et al., 1994; Agris, 1996; Nobles et al., 2002). However, the precise concentrations of Mg^2+^ ions in *T. thermophilus* cells are unknown. K^+^ ions are also important for correct folding of tRNA. The concentrations of K^+^ ions in the cells of several thermophilic archaea are reported to be extremely high (>700 mM) (Hensel and Konig, 1988). However, the intracellular concentrations of K^+^ ions of *T. thermophilus* have not been reported. The concentrations of Mg^2+^ and K^+^ ions may change depending on the growth environment.

## Perspective

2018 marked the 50th anniversary year of the first isolation of *T. thermophilus*. In these past 50 years, *T. thermophilus* has been studied as a model organism that can adapt to extremely high temperatures. In this regard, the modifications in tRNA have been studied mainly from the viewpoint of the stabilization of tRNA structure at high temperatures. In particular, the role of m^5^s^2^U54 in tRNA has been clarified, the genes responsible for almost all tRNA modifications in *T. thermophilus* have been annotated and the regulatory network between modified nucleosides and tRNA modification enzymes has been identified. Furthermore, numerous tRNA modification enzymes from *T. thermophilus* have been used in structural studies (reviewed in Hori et al., 2018).

Nevertheless, several enigmas remain, even today. For example, the functions of tRNA modifications in the anticodon-loop at high temperatures have not been studied in detail. Studies on anticodon-loop modifications are difficult because *in vitro* protein synthesis of *T. thermophilus* does not work effectively at high temperatures (>75°C). Although there have been many attempts to synthesize proteins at high temperatures using a *T. thermophilus* cell-free translation system (Ohno-Iwashita et al., 1975; Uzawa et al., 1993a,b; Zhou et al., 2012), it remains difficult to monitor protein synthesis at 80°C. As a result, our knowledge about the effects of tRNA modifications on codon-anticodon interactions, on maintenance of reading frame and on dynamics of tRNA on ribosome at high temperatures is limited. Furthermore, recent studies on mesophiles reported that tRNA modifications occur in response to environmental stresses or function as stress resistance factors (Nawrot et al., 2011; Preston et al., 2013; Endres et al., 2015; Jaroensuk et al., 2016; Campos Guillen et al., 2017). As yet, however, there are no studies from this viewpoint for *T. thermophilus*. To understand the roles of tRNA modifications in totality, further studies will be required.

## Author Contributions

HH determined the concept of this review and prepared the manuscript.

## Conflict of Interest Statement

The author declares that the research was conducted in the absence of any commercial or financial relationships that could be construed as a potential conflict of interest.

## Footnotes

1 http://dna.brc.riken.jp/ja/thermus

2 https://www.ncbi.nlm.nih.gov/gds/
